# Characterizing Direct-to-Consumer Stem Cell Businesses in the Southwest United States

**DOI:** 10.1016/j.stemcr.2019.07.001

**Published:** 2019-08-01

**Authors:** Emma K. Frow, David A. Brafman, Anna Muldoon, Logan Krum, Paige Williams, Bryson Becker, John P. Nelson, Ashley Pritchett

**Affiliations:** 1 School for the Future of Innovation in Society, Arizona State University, Tempe, AZ 85287, USA; 2 School of Biological & Health Systems Engineering, Arizona State University, Tempe, AZ 85287, USA; 3 School of Life Sciences, Arizona State University, Tempe, AZ 85287, USA; 4 School of Human Evolution and Social Change, Arizona State University, Tempe, AZ 85287, USA; 5 College of Health Solutions, Arizona State University, Phoenix, AZ 85004, USA

**Keywords:** stem cell clinics, direct-to-consumer treatments, FDA, policy, regulation

## Abstract

There are currently hundreds of businesses across the United States offering direct-to-consumer stem cell treatments that have not been through regulatory approval by the Food and Drug Administration (FDA). Here, we provide a detailed characterization of nearly 170 stem cell businesses operating in the Southwest United States. We draw specific attention to two as-yet understudied facets of these businesses. First, we identify differences in the degree to which a given business focuses their practice on stem cell treatments. Second, we compare the stated expertise of the care providers in stem cell businesses with the range of conditions they purport to treat. These findings deepen our knowledge of the growing industry around unapproved stem cell treatments, and are used here to offer suggestions for how the FDA might target its resources with respect to regulatory oversight.

## Highlights

• We characterize 169 direct-to-consumer stem cell businesses in the southwest US
• We show differences in degree of clinic focus on providing stem cell treatments
• We compare care provider expertise with the stem cell-based interventions they offer
• We offer suggestions for regulatory oversight of direct-to-consumer stem cell businesses

## Teaser

Frow and colleagues provide a detailed characterization of 169 direct-to-consumer stem cell businesses operating in the Southwest US. They identify differences in the degree to which individual clinics focus on providing stem cell treatments, and compare physician expertise with the types of conditions treated. These findings are used to offer suggestions for regulatory oversight of direct-to-consumer stem cell businesses.

## Introduction

Recent years have seen growing attention paid to the rapid rise of clinics offering direct-to-consumer stem cell treatments that have not gone through approval by the Food and Drug Administration (FDA). These developments are being tracked by the academic community in various ways, including studies tracing the rise of these clinics (Knoepfler and Turner, 2018), characterizing the conditions they offer to treat (Lau et al., 2008, Turner and Knoepfler, 2016) and their marketing practices (Knoepfler, 2017, Sipp et al., 2017), and analyzing press coverage of celebrities who have had experimental stem cell treatments (Rachul and Caulfield, 2015).

Our current study draws on and extends previous research by offering more a granular and detailed characterization of a subset of clinics operating in the United States. We focus on the six Southwest states (Arizona, California, Colorado, Nevada, New Mexico, and Utah). Together, these six states capture approximately one-third of the total number of businesses in the United States identified by Turner and Knoepfler (2016), and include four of the seven “hot-spot” cities they note (Beverly Hills, Los Angeles, Phoenix, and Scottsdale).

In this paper, we draw attention to two as-yet understudied facets of stem cell clinics. First, we identify differences in the degree to which individual clinics orient their practices around stem cell treatments. Second, we explore the issue of medical expertise, comparing the specialties of care providers practicing at clinics focused solely on stem cell treatments with the conditions they purport to treat. We suggest that understanding the links between provider expertise and stem cell treatments could provide useful information for patients and regulators.

## Results

### Stem Cell Types and Conditions Treated

**Figure 1:**
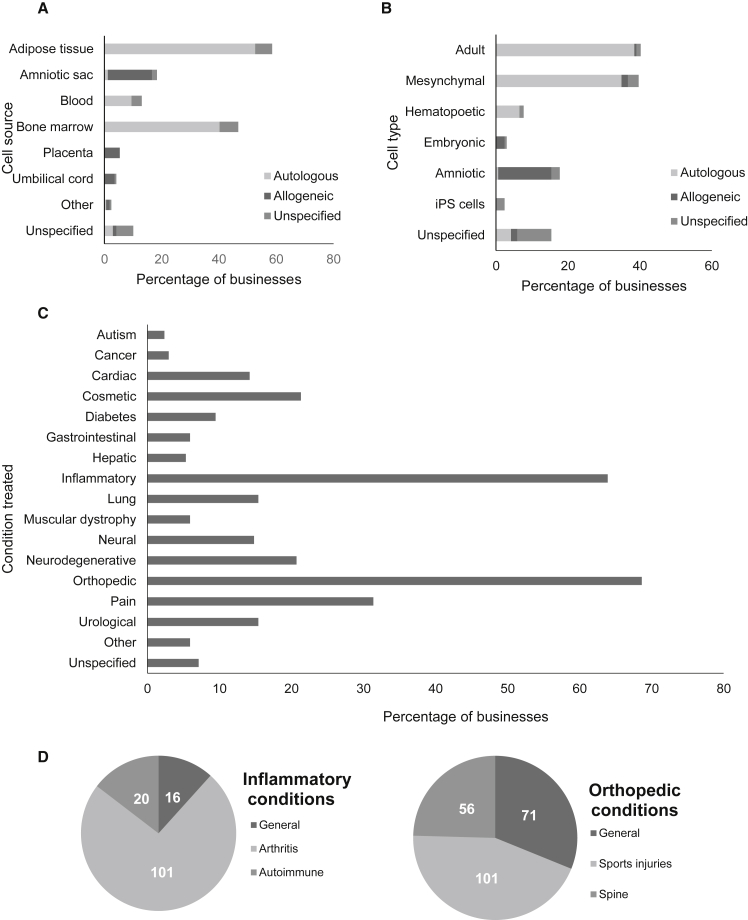
Stem Cell Types and Conditions Treated (A) Sources of stem cells used by stem cell businesses. (B) Type of stem cell used. For (A) and (B), some businesses indicate use of more than one cell type or cell source. (C) Types of medical conditions treated by stem cell businesses (see Table S1 for lists of specific conditions included within each category). (D) Pie charts detailing the breakdown of inflammatory and orthopedic conditions treated. A single business may offer treatment for more than one category of medical condition (see Figure 2C). Data presented reflect information explicitly stated on the website of a given stem cell business (n = 169).

Our results broadly corroborate the findings of Turner and Knoepfler (2016), with adipose tissue being the stated source of stem cells for nearly two-thirds of the stem cell businesses, and bone marrow used by almost half (Figure 1A). The majority of stem cell businesses use adult stem cells, using varied terminology including “adult,” “mesenchymal,” and “hematopoietic” stem cells. We report the terms used by the businesses themselves, acknowledging that the type of cell being advertised is not necessarily consistent with current scientific terminology. About 20% of businesses offer amniotic cells (Figure 1B), an allogeneic stem cell type that has seen increased marketing since 2012 (Knoepfler and Turner, 2018). Preparations derived from adipose tissue, bone marrow, and blood are typically used for autologous treatments. About 25% (45/169) of businesses offer more than one source of stem cells, and 40% (70/169) offer more than one cell type in marketing their treatments.

We recorded each medical condition that businesses purport to treat with stem cells, and condensed the resultant list into 11 broad categories (Table S1). By far the most commonly treated conditions were orthopedic and inflammatory conditions, followed by pain, cosmetic, and neurodegenerative conditions (Figure 1C). While our analysis corroborates the findings of Turner and Knoepfler (2016) regarding the marketing of orthopedic conditions, we identify a much higher percentage of clinics offering to treat inflammatory conditions. This could indicate a geographical trend toward the treatment of inflammatory conditions in the southwestern states, or more generally an increase in the percentage of clinics offering to treat inflammatory conditions since 2016. Further examination is under way of the specific evidence that these businesses present to support their use of a given cell type or cell source for the treatment of a particular condition, but preliminary analysis suggests that these applications are largely “unproven” as defined by scientific norms and professional academic societies (Daley et al., 2016, Sipp et al., 2017, Srivastava et al., 2016).

### Business Models

**Figure 2:**
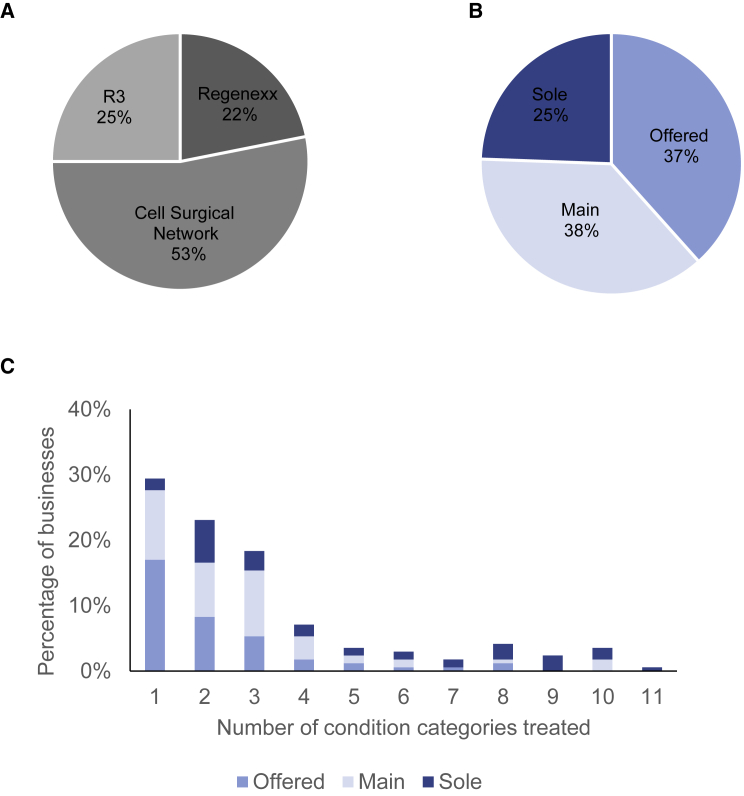
Insights into the Business Models of Stem Cell Businesses (A) Stem cell businesses belonging to a franchise operation (n = 34). (B) Degree of business focus on stem cell treatments. Each stem cell business was categorized according to whether stem cells were an offered treatment (among many other treatment types), a main focus of the business, or the sole treatment offered (n = 169). (C) Number of condition categories (shown in Figure 1C) treated by each stem cell business (n = 163, as not all businesses list conditions treated). Businesses are broken down by degree of focus on stem cells. Data presented are based on information stated on clinic websites.

It is not unusual for a given stem cell business to run clinics in multiple locations; we identify that 26% of stem cell businesses in the Southwest operate out of more than one location. In total, 20% of businesses indicated an affiliation to one of three franchises: Cell Surgical Network (headquartered in California, advertising primarily autologous treatments with adipose tissue for treating a wide range of conditions), Regenexx/Regenerative Sciences (now headquartered in Iowa, focused on the use of autologous bone marrow-derived preparations to treat orthopedic conditions), and R3 (headquartered in Arizona, advertising primarily amniotic and umbilical tissue treatments for a wide range of conditions) (Figure 2A). Stem cell franchises offer care providers access to equipment and protocols, provide centralized online information and marketing, and lend a recognizable name across clinics.

All three of the franchises we identified have locations across the country, but 40% of their listed clinics are located in the Southwest. This said, the low overall percentage of businesses in the Southwest affiliating with a franchise (20%) suggests that the barriers to entry for a stem cell business are relatively low. It seems realistic to suggest that the Southwest is representative of the situation across the United States as a whole; together, these three franchises list just over 200 clinics across the country (as of May 2019), and the number of stem cell clinics operating in the United States was estimated at 716 in May 2017 (Turner, 2018) and is likely to be even higher now. That 70%–80% of stem cell businesses are operating outside of franchises may limit the effectiveness of pursuing regulatory action by targeting franchises. This said, the FDA is currently seeking a permanent injunction against the Cell Surgical Network for numerous violations of good manufacturing and tissue practice, and for marketing products without FDA approval (United States of America v. California Stem Cell Treatment Center Inc, 2018), and previously won a court case against Regenerative Sciences for violating manufacturing and labeling requirements (United States of America v. Regenerative Sciences, LLC, 2014).

Intriguingly, approximately one-third (51/169) of stem cell businesses share a physical address with another medical or cosmetic clinic. These co-located businesses typically maintain different websites, phone numbers, and contact emails, but at least 40% of them share care providers (for the other 60% of clinics, the information supplied on the websites did not make it possible to identify whether there was overlap in care providers). The rationale for a care provider to maintain a stem cell business distinct from a medical practice is a matter of speculation, but this division may financially and legally insulate the two practices.

During the data collection process, we observed that businesses differ in the degree to which stem cell treatments are presented as central to their practice. Based on this, we subjectively divided businesses into one of three categories based on their degree of focus on stem cell treatments: stem cells as one treatment offered among many (37%), stem cells as a main treatment offered (38%), and stem cells as the sole focus of treatment (25%) (Figure 2B). The predominant model for stem cell treatments is thus not in the form of bespoke clinics, but rather as one type of treatment offered by businesses that may specialize in particular medical conditions (e.g., orthopedic conditions) or types of intervention (e.g., cosmetic surgery).

Sole-focus businesses do show some different patterns from businesses offering stem cell interventions as one of several types of treatment. For example, while 20% of stem cell businesses are part of franchises, this number reaches 38% for sole-focus businesses. Furthermore, 45% of sole-focus businesses are co-located with another medical practice.

Across the Southwest, two-thirds of clinics offer to treat more than one category of medical condition with stem cells. We identify a majority (71%) of clinics treating 1–3 categories of medical conditions, and only 1% that treat conditions from all of the 11 condition categories we identified (Figure 2C). Businesses focused solely on stem cells treat medical conditions across the full range of medical-condition categories. Of the 44 stem cell businesses in the Southwest that market treatments for four or more categories of medical conditions, those focused solely on stem cells are overrepresented (21/44, Figure 2C). Tracking the number of conditions treated by a given business can assist with identifying violations of FDA guidance on homologous use of human cells, tissues, and cellular and tissue-based products, which identifies the use of a single type of stem cell treatment for multiple medical conditions as an indication that the treatment might not adhere to the homologous use criterion (Food and Drug Administration, 2017).

### Sole-Focus Businesses: Medical Expertise and Treatment Types

**Figure 3:**
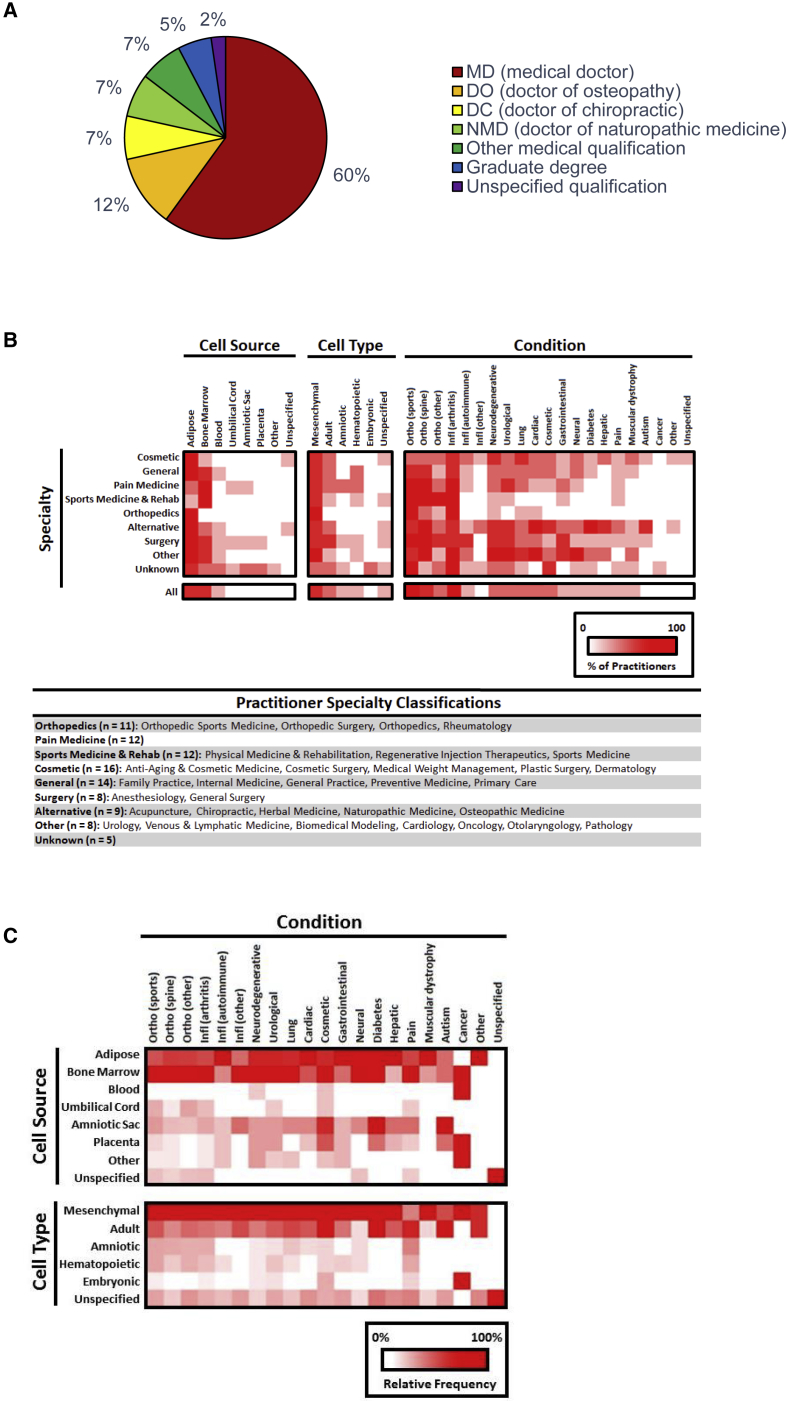
Medical Expertise and Stem Cell Treatments Data reflect sole-focus businesses only. (A) Breakdown of employees by qualification (n = 130). “Other medical qualification” category includes Doctor of Podiatric Medicine (n = 1), physical therapists (n = 1), physician assistants (n = 5), and nurse practitioners (n = 2). Seven individuals listed non-medical graduate degrees, and a further three did not specify their qualifications. Two of the sole-focus businesses did not provide any information regarding practice employees or care providers. (B) Relationship between practitioner specialty and stem cell source, stem cell type, and medical conditions treated. Data are shown as the percentage of practitioners with an indicated specialty who use a specific stem cell source or stem cell type as well as treat a specific medical condition. (C) Relationship between stem cell source/type and medical condition treated. Data are shown as the column-normalized, relative frequency with which a specific condition is treated with a particular stem cell source/type. Analysis is based on information stated on stem cell business websites. A single business may use more than one cell type and cell source, and offer to treat more than one condition. Multiple care providers may also practice within a given business.

For clinics offering stem cells as one of many possible treatment options, it is typically impossible to determine from their websites which of the care providers might administer stem cell treatments. To examine the relationship between medical expertise and stem cell treatments, we narrowed our subsequent analysis to those businesses focused exclusively on stem cells. Of the 130 employees listed on the websites of sole-focus clinics in the Southwest, 60% have MD qualifications (Figure 3A). Other medical qualifications include Doctor of Osteopathy (12%), Doctor of Chiropractic (7%), and Doctor of Naturopathic Medicine (NMD, 7%). Interestingly, eight of the nine practicing NMDs in the Southwest are based in Arizona, and each received their NMD degree from the same naturopathic institution.

In addition to capturing the types of degrees held by care providers working in stem cell businesses, we worked to identify their medical specialties by systematically gathering their self-reported information regarding any board certifications and professional memberships. We observed that care providers of all specialties market the treatment of arthritis, spine, and sports injuries (Figure 3B). Specialists in orthopedics and sports medicine and rehabilitation were more likely to restrict stem cell treatments to those conditions related to their medical specialties (orthopedic conditions and arthritis). Providers listing specialties in cosmetic or alternative medicine were more likely to treat medical conditions across the full range of categories identified (with the exception of cancer).

As a whole, sole-focus businesses did not show different trends from the full set of businesses in the overall frequency of cell source or cell type used, or conditions treated (compare the “All” row in Figure 3B with Figures 1A–1C). Looking at the relationship between cell source and conditions treated, we identify that adipose tissue and bone marrow are used to treat the widest range of conditions (Figure 3C, upper panel). Across sole-focus businesses, any given condition appears to be treated using cells from multiple sources, and any given cell type is used to treat multiple conditions. Cosmetic, orthopedic, and inflammatory conditions are treated using cells from the widest range of sources, while treatments for muscular dystrophy are restricted to adipose and bone marrow sources. Similar patterns emerge when considering cell type rather than cell source (Figure 3C, lower panel), with mesenchymal and adult stem cells being used to treat every condition category. These heatmaps highlight the still highly experimental and diffuse landscape for stem cell treatments, with little convergence across clinics regarding which cell preparations might be best suited to treating different medical conditions.

## Discussion: Policy Implications

Recent years have seen repeated calls for the FDA to take action against clinics marketing direct-to-consumer stem cell interventions (e.g., Turner and Knoepfler, 2016). Our analysis of stem cell businesses in the Southwest United States identifies several specific factors that could assist the FDA, state medical licensing boards, and prospective patients in prioritizing among businesses and care providers that warrant closer scrutiny.

First, we suggest that the 25% of stem cell businesses focusing exclusively on stem cell treatments be prioritized for closer attention. For clinics that offer stem cells as one of many treatment options, it can be difficult to identify which portion of their business is stem cell related.

Second, clinics purporting to use adipose tissue as a source of stem cells are also potential targets for scrutiny, as these procedures are likely to be out of compliance with the final FDA guidance adopted in 2017. With adipose tissue now classified as a structural tissue, clinics may be required to pursue FDA approval for treatments making use of this cell source. It remains to be seen whether stem cell clinics begin to move away from adipose tissue, and toward bone marrow, amniotic, or other stem cell sources, in light of this classification.

Third, we advocate greater scrutiny of those businesses offering to treat multiple condition types with stem cells. Approximately 30% of the clinics in the Southwest offer to treat four or more types of medical conditions, which may indicate a greater likelihood of violating FDA guidance on homologous use.

Fourth, the patterns we identify between medical expertise and conditions treated with stem cells can be leveraged to identify clinics for further scrutiny. Specialists in orthopedics and sports medicine and rehabilitation were more likely to restrict stem cell treatments to conditions falling within their specialty area. We identify specialists in cosmetic and anti-aging medicine as treating the widest variety of medical conditions, and suggest that practitioners with these specialties be prioritized for review by both FDA and state medical licensing boards. We also recommend that the helpful guide for patients published by the International Society for Stem Cell Research (2015) consider including a question about whether a practitioner's stated medical credentials are well suited to the medical condition for which a patient is seeking treatment.

In summary, our detailed characterization of stem cell businesses in the Southwest United States offers new insights into the practices of direct-to-consumer stem cell clinics, and identifies a concrete set of variables that may be of assistance to regulatory bodies and patients trying to make sense of this rapidly growing market in the United States.

## Experimental Procedures

We gathered publicly available, online material relating to stem cell businesses and their practitioners, including information relating to the types and sources of stem cells used, the conditions treated, the franchise status of the business, and the self-reported qualifications of their medical providers. We began by characterizing clinics already identified by Turner and Knoepfler (2016), who listed 128 businesses operating across 207 locations in the six Southwest states. By expanding our internet search terms to include US State names and large cities in the Southwest, we identified an additional 41 businesses and 33 clinic locations in these six states, for a total of 169 businesses across 238 clinic locations (Table S2). We do not claim to have identified a comprehensive list of clinics operating in the Southwest, but our findings suggest that the estimate by Turner and Knoepfler (2016) was conservative, and/or that the number of clinics increased significantly between the end of their data collection (February 2016) and ours (August 2017). Indeed, a revised estimate from Turner (2018) suggests 716 clinics operating across the United States as of May 2017, compared with 570 in February 2016.

There is great heterogeneity in the terminology and type of information presented on the websites of stem cell businesses. To this end, we developed a uniform classification system and categorization of stem cell types and sources, medical conditions, and practitioner specialties presented across the websites. Table S1 groups all medical conditions treated by stem cell businesses into a set of broader categories. An overview of all the data collected is presented for each business, in de-identified form, as Table S3.

## Author Contributions

Project Conceptualization and Supervision, E.K.F. and D.A.B.; Data Collection, A.M., E.K.F., L.K., P.W., B.B., J.P.N., and A.P.; Data Curation and Analysis, A.M. and L.K.; Writing – Original Draft, A.M. and E.K.F.; Writing – Review & Editing, D.A.B. and E.K.F.; Figure Preparation, A.M., D.A.B., and E.K.F.

## Supplemental Information

Document S1. Supplemental Experimental Procedures and Tables S1 and S2

Table S3. Anonymized Overview of the Raw Data Collected for Each of the 169 Stem Cell Businesses Analyzed in This Article The data presented here were used for the preparation of Figures 1–3.

Document S2. Article plus Supplemental Information
