# Supplementary material for: Characterizing Direct-to-Consumer Stem Cell Businesses in the Southwest United States
Source: Stem Cell Reports. 2019 Aug 1;13(2):247–53. doi: 10.1016/j.stemcr.2019.07.001 (PMC6700498; doi:10.1016/j.stemcr.2019.07.001)
Supplement: Document S1. Supplemental Experimental Procedures and Tables S1 and S2 [file mmc1.pdf]

Stem Cell Reports, Volume *13*

Supplemental Information

Characterizing Direct-to-Consumer Stem Cell Businesses in the South-west United States

Emma K. Frow, David A. Brafman, Anna Muldoon, Logan Krum, Paige Williams, Bryson Becker, John P. Nelson, and Ashley Pritchett

Supplemental Information

Table S1. List of medical conditions treated with stem cells across the businesses characterized (related to Fig.1C).

**Condition Category**	**Conditions included in the category**
Autism	
Cancer	Breast cancer
	Prostate cancer
Cardiac	Cardiovascular problems
	Chronic heart failure
	Ischemic cardiomyopathy
	Ischemic stroke
	Myocardial infarction
	peripheral artery disease
	refractory angina
Cosmetic	anti-aging
	breast augmentation
	breast reconstruction
	buttock augmentation
	cutaneous photo-aging
	Facelift
	facial rejuvenation
	hair rejuvenation
	Skin
Diabetes	
Gastrointestinal (GI)	Crohn's disease
	ulcerative colitis
Hepatic	fatty liver / liver insufficiency
	Hepatitis
	auto-immune hepatitis
Inflammatory	SI joint inflammation
	Facet joint syndrome
(sub-category) Arthritis	basal joint arthritis
	carpometacarpal arthritis
	degenerative osteoarthritis
	Osteoarthritis
	rheumatoid arthritis
	spinal arthritis
	sub-talar arthritis
(sub-category) Autoimmune	Alopecia

	Lupus
	relapsing polychondritis
Lung	Asthma
	COPD
Muscular dystrophy	
Neural	cognitive impairment
	CIDP
	diabetic neuropathy
	Dysautonomia
	myasthemia gravis
	occipital neuralgia
	optic neuritis
	peripheral neuropathy
	Radiculopathy
	Sciatica
	Stroke
	traumatic brain injury
Neurodegenerative conditions	ALS
	Alzheimer's
	cerebral palsy
	Huntington's
	Multiple sclerosis
	Parkinsons
Orthopedic	AC joint separation
	avascular osteonecrosis
	Baker's cyst
	Bursitis
	Bunion
	carpal/tarsal tunnel syndrome
	Dupuytren's contracture
	Osteoporosis
	pinched nerve/radiating pain
	plantar fasciitis
	spinal muscular atrophy
	Spur
	thoracic outlet syndrome
	Tumor
(sub-category) Sports injuries	Achilles injuries

	ACL
	Fractures
	Golfer's elbow
	Instability
	Meniscus
	osteochondral lesions
	patellar, patellofemoral syndrome
	post-surgery
	recurrent dislocations
	rotator cuff tendonitis
	runner's knee
	Sclerodoma
	Sprains
	tendonitis, tendonopathy
	tennis elbow
	TFCC tear
	trigger finger
(sub-category) Spine	annular tear
	Deformity
	degenerative disk disease
	failed back surgery
	herniated disk
	Scoliosis
	spondylosis; spondylolisthesis
	spinal stenosis
	Stenosis
Pain	General
	headaches/migraines
	post-surgery
	Whiplash
Urological	erectile dysfunction
	interstitial cystitis
	peyronies disease
	bladder conditions
	kidney conditions

Table S2. Comparison of number of stem cell businesses & clinics identified by Turner and Knoepfler (2016) and those identified in the current study (related to Experimental Procedures).

	**Current study**		**Turner & Knoepfler (2016)**	
**State**	**Businesses**	**Clinics**	**Businesses**	**Clinics**
**Arizona**	34	47	27	36
**California**	79	105	71	113
**Colorado**	28	41	19	37
**New Mexico**	6	8	1	2
**Nevada**	11	15	5	7
**Utah**	11	22	5	10
**TOTAL**	169	238	128	205

Table S3. Data summary for direct-to-consumer stem cell businesses in the Southwest US (related to Experimental Procedures). State locations are provided for each business, but specific business names are not identified.

See Excel file for Table S3.

Supplemental Experimental Procedures

Online data collection

Stem cell businesses were identified through state-by-state internet searches between June 2016 and August 2017. The list of businesses provided by Turner and Knoepfler (2016) was used to create an initial list, and was then expanded through independent searches for businesses in specific states and regions in the Southwest US. Specific search terms used are listed at the end of this document.

Information was collected from the public sections of stem cell business websites and inputted into an Excel spreadsheet. For each characterized stem cell business, screenshots were taken of each webpage that mentioned stem cells (using Snagit software), to construct an archive for future reference. After the initial data collection, a randomized 25% of the clinics characterized by each researcher was cross-checked by a different member of the research team, to ensure consistency in data collection practices across researchers. Any discrepancies in data collection were flagged and discussed collectively, and a consensus data entry agreed upon.

The data collected reflects the public presentation of a stem cell business; it is assumed to be accurate, but is not guaranteed to represent the actual practices of a given business. For example, a given clinic might not list all the conditions they treat with stem cells, or might not in practice treat all the conditions that they list online.

Condition Categories

A complete list of medical conditions treated with stem cells was compiled all from stem cell business websites characterized, and the research team worked collectively to collapse this list into the 11 broad categories listed in Table S1.

Medical Specialties

The care providers listed for each stem cell business were copied into the data collection spreadsheets, together with any specialty and/or board certification information listed on the business website. For any providers who did not list information regarding their professional qualifications, Google searches were undertaken to identify any specialties, board certifications, or professional association memberships. If a given practitioner made information available on a university or other clinic website, this was used. For those who did not, searches were conducted to identify available biographies from conferences, fellowships, or professional societies. If no listing for a care provider could be found or no names were provided on a clinic's website, the provider was listed as of "unknown" expertise.

Once identified, medical specialties were grouped into related categories for analysis (see Fig. 3B). Some specialties were combined for brevity. For example, "anti-aging," "anti-aging and cosmetic medicine," and "cosmetic medicine" were combined into "anti-aging and cosmetic medicine". "General practice" and "general medicine" were combined into "general practice."

Stem Cell Business Search Terms

Stem cell clinics Arizona
Stem cell treatment clinic Southern California
Northern California stem cell treatment center
Norcal regenerative cell treatment
Bay Area stem cell treatment center
Colorado stem cell clinics
Stem cell clinics AND New Mexico
Stem cell clinics and NM
Stem cell clinics in New Mexico
Adipose tissue AND New Mexico
Stem cell clinics Nevada
Stem cell clinics of Nevada
Small stem cell clinics in Nevada
Stem cell clinics Utah
Health AND stem cells
