# Supplementary material for: Characterizing Direct-to-Consumer Stem Cell Businesses in the Southwest United States
Source: Stem Cell Reports. 2019 Aug 1;13(2):247–53. doi: 10.1016/j.stemcr.2019.07.001 (PMC6700498; doi:10.1016/j.stemcr.2019.07.001)
Supplement: Document S2. Article plus Supplemental Information [file mmc3.pdf]

Stem Cell Reports
Report
OPEN ACCESS

Characterizing Direct-to-Consumer Stem Cell Businesses in the Southwest United States

Emma K. Frow,[1,2,6,*] David A. Brafman,[2,6,*] Anna Muldoon,[1] Logan Krum,[3] Paige Williams,[4] Bryson Becker,[3] John P. Nelson,[1] and Ashley Pritchett[5]
[1]School for the Future of Innovation in Society, Arizona State University, Tempe, AZ 85287, USA
[2]School of Biological & Health Systems Engineering, Arizona State University, Tempe, AZ 85287, USA
[3]School of Life Sciences, Arizona State University, Tempe, AZ 85287, USA
[4]School of Human Evolution and Social Change, Arizona State University, Tempe, AZ 85287, USA
[5]College of Health Solutions, Arizona State University, Phoenix, AZ 85004, USA
[6]Co-first author
*Correspondence: emma.frow@asu.edu (E.K.F.), david.brafman@asu.edu (D.A.B.)
https://doi.org/10.1016/j.stemcr.2019.07.001

SUMMARY

There are currently hundreds of businesses across the United States offering direct-to-consumer stem cell treatments that have not been through regulatory approval by the Food and Drug Administration (FDA). Here, we provide a detailed characterization of nearly 170 stem cell businesses operating in the Southwest United States. We draw specific attention to two as-yet understudied facets of these businesses. First, we identify differences in the degree to which a given business focuses their practice on stem cell treatments. Second, we compare the stated expertise of the care providers in stem cell businesses with the range of conditions they purport to treat. These findings deepen our knowledge of the growing industry around unapproved stem cell treatments, and are used here to offer suggestions for how the FDA might target its resources with respect to regulatory oversight.

INTRODUCTION

Recent years have seen growing attention paid to the rapid rise of clinics offering direct-to-consumer stem cell treatments that have not gone through approval by the Food and Drug Administration (FDA). These developments are being tracked by the academic community in various ways, including studies tracing the rise of these clinics (Knoepfler and Turner, 2018), characterizing the conditions they offer to treat (Lau et al., 2008; Turner and Knoepfler, 2016) and their marketing practices (Knoepfler, 2017; Sipp et al., 2017), and analyzing press coverage of celebrities who have had experimental stem cell treatments (Rachul and Caulfield, 2015).

Our current study draws on and extends previous research by offering more a granular and detailed characterization of a subset of clinics operating in the United States. We focus on the six Southwest states (Arizona, California, Colorado, Nevada, New Mexico, and Utah). Together, these six states capture approximately one-third of the total number of businesses in the United States identified by Turner and Knoepfler (2016), and include four of the seven "hot-spot" cities they note (Beverly Hills, Los Angeles, Phoenix, and Scottsdale).

In this paper, we draw attention to two as-yet understudied facets of stem cell clinics. First, we identify differences in the degree to which individual clinics orient their practices around stem cell treatments. Second, we explore the issue of medical expertise, comparing the specialties of care providers practicing at clinics focused solely on stem cell treatments with the conditions they purport to treat. We suggest that understanding the links between provider expertise and stem cell treatments could provide useful information for patients and regulators.

RESULTS

Stem Cell Types and Conditions Treated

Our results broadly corroborate the findings of Turner and Knoepfler (2016), with adipose tissue being the stated source of stem cells for nearly two-thirds of the stem cell businesses, and bone marrow used by almost half (Figure 1A). The majority of stem cell businesses use adult stem cells, using varied terminology including "adult," "mesenchymal," and "hematopoietic" stem cells. We report the terms used by the businesses themselves, acknowledging that the type of cell being advertised is not necessarily consistent with current scientific terminology. About 20% of businesses offer amniotic cells (Figure 1B), an allogeneic stem cell type that has seen increased marketing since 2012 (Knoepfler and Turner, 2018). Preparations derived from adipose tissue, bone marrow, and blood are typically used for autologous treatments. About 25% (45/169) of businesses offer more than one source of stem cells, and 40% (70/169) offer more than one cell type in marketing their treatments.

We recorded each medical condition that businesses purport to treat with stem cells, and condensed the resultant

This is an open access article under the CC BY license (http://creativecommons.org/licenses/by/4.0/).

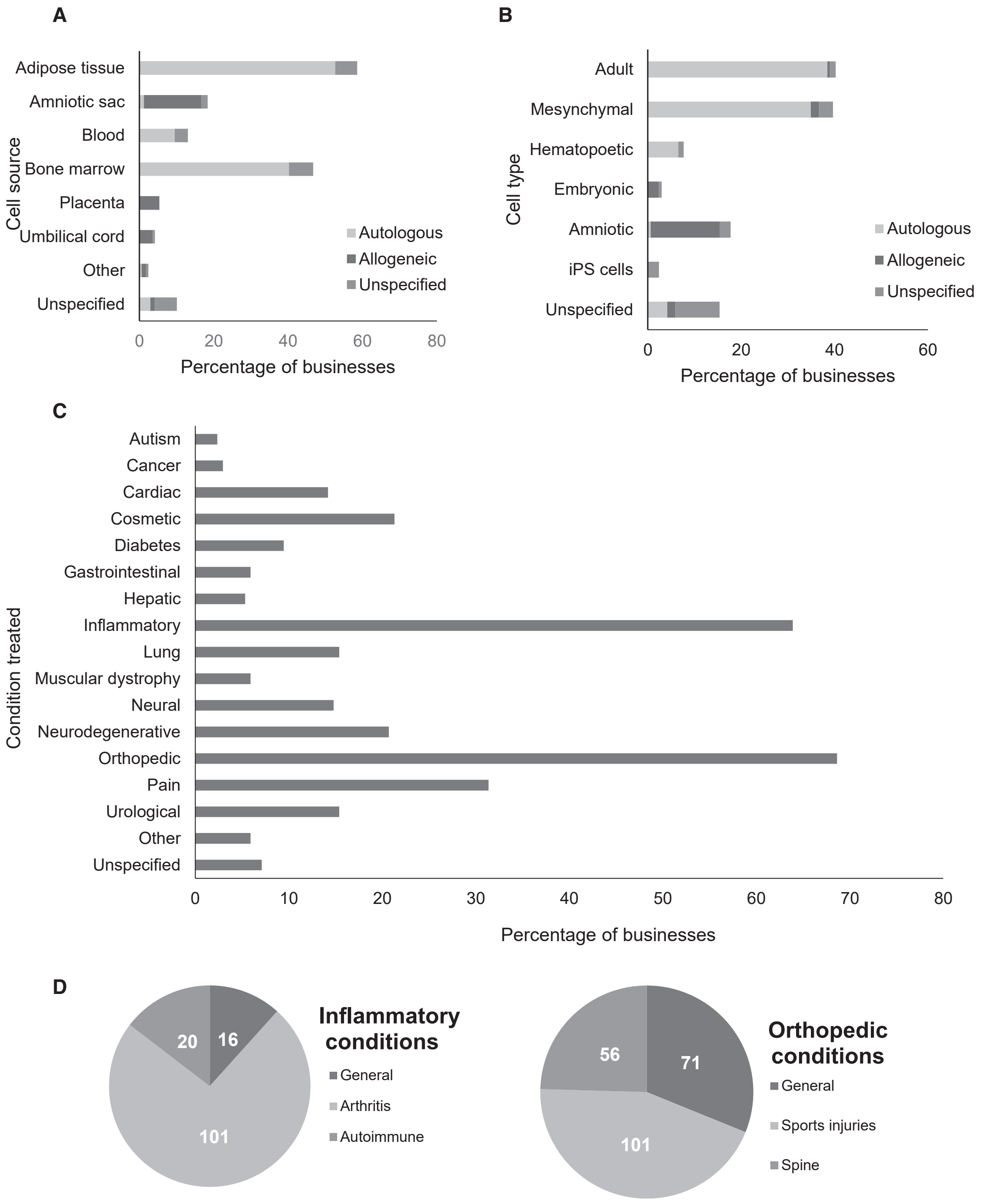

Figure 1. Stem Cell Types and Conditions Treated

(A) Sources of stem cells used by stem cell businesses.

(B) Type of stem cell used. For (A) and (B), some businesses indicate use of more than one cell type or cell source.

(C) Types of medical conditions treated by stem cell businesses (see Table S1 for lists of specific conditions included within each category).

(D) Pie charts detailing the breakdown of inflammatory and orthopedic conditions treated. A single business may offer treatment for more than one category of medical condition (see Figure 2C). Data presented reflect information explicitly stated on the website of a given stem cell business (n = 169).

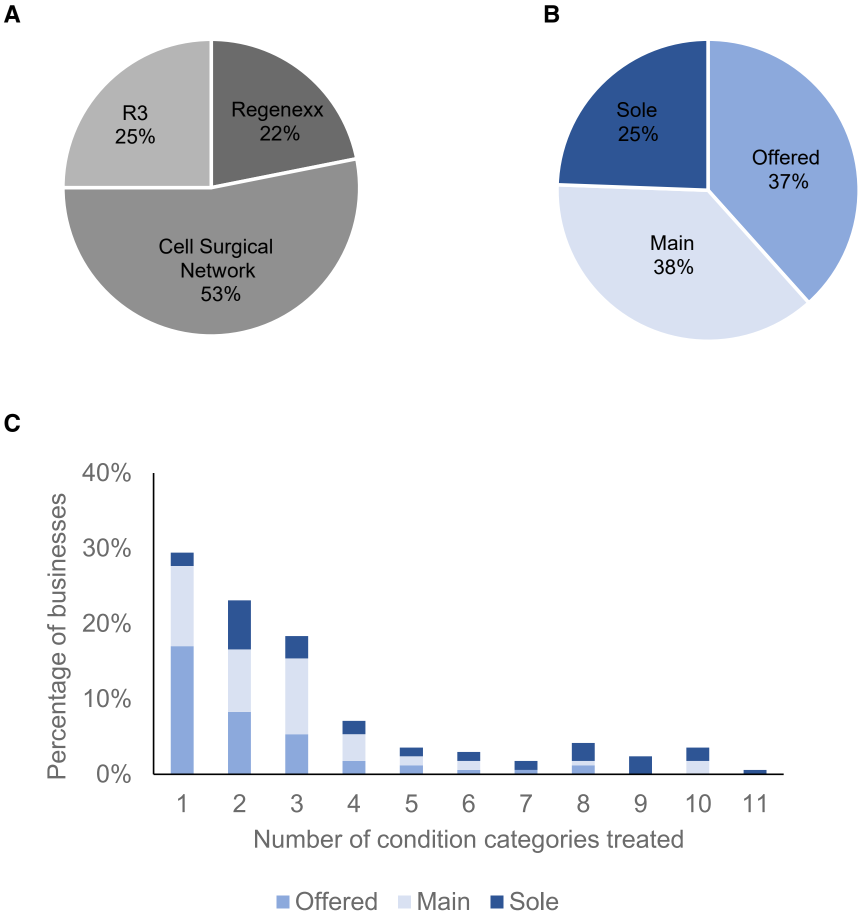

Figure 2. Insights into the Business Models of Stem Cell Businesses

(A) Stem cell businesses belonging to a franchise operation (n = 34).

(B) Degree of business focus on stem cell treatments. Each stem cell business was categorized according to whether stem cells were an offered treatment (among many other treatment types), a main focus of the business, or the sole treatment offered (n = 169).

(C) Number of condition categories (shown in Figure 1C) treated by each stem cell business (n = 163, as not all businesses list conditions treated). Businesses are broken down by degree of focus on stem cells. Data presented are based on information stated on clinic websites.

list into 11 broad categories (Table S1). By far the most commonly treated conditions were orthopedic and inflammatory conditions, followed by pain, cosmetic, and neurodegenerative conditions (Figure 1C). While our analysis corroborates the findings of Turner and Knoepfler (2016) regarding the marketing of orthopedic conditions, we identify a much higher percentage of clinics offering to treat inflammatory conditions. This could indicate a geographical trend toward the treatment of inflammatory conditions in the southwestern states, or more generally an increase in the percentage of clinics offering to treat inflammatory conditions since 2016. Further examination is under way of the specific evidence that these businesses present to support their use of a given cell type or cell source for the treatment of a particular condition, but preliminary analysis suggests that these applications are largely “unproven” as defined by scientific norms and professional academic societies (Daley et al., 2016; Sipp et al., 2017; Srivastava et al., 2016).

Business Models

It is not unusual for a given stem cell business to run clinics in multiple locations; we identify that 26% of stem cell businesses in the Southwest operate out of more than one location. In total, 20% of businesses indicated an affiliation to one of three franchises: Cell Surgical Network (headquartered in California, advertising primarily autologous treatments with adipose tissue for treating a wide range of conditions), Regenexx/Regenerative Sciences (now headquartered in Iowa, focused on the use of autologous bone marrow-derived preparations to treat orthopedic conditions), and R3 (headquartered in Arizona, advertising primarily amniotic and umbilical tissue treatments for a wide range of conditions) (Figure 2A). Stem cell franchises offer care providers access to equipment and protocols, provide centralized online information and marketing, and lend a recognizable name across clinics.

All three of the franchises we identified have locations across the country, but 40% of their listed clinics are located in the Southwest. This said, the low overall percentage of businesses in the Southwest affiliating with a franchise (20%) suggests that the barriers to entry for a stem cell business are relatively low. It seems realistic to suggest that the Southwest is representative of the situation across the United States as a whole; together, these three franchises list just over 200 clinics across the country (as of

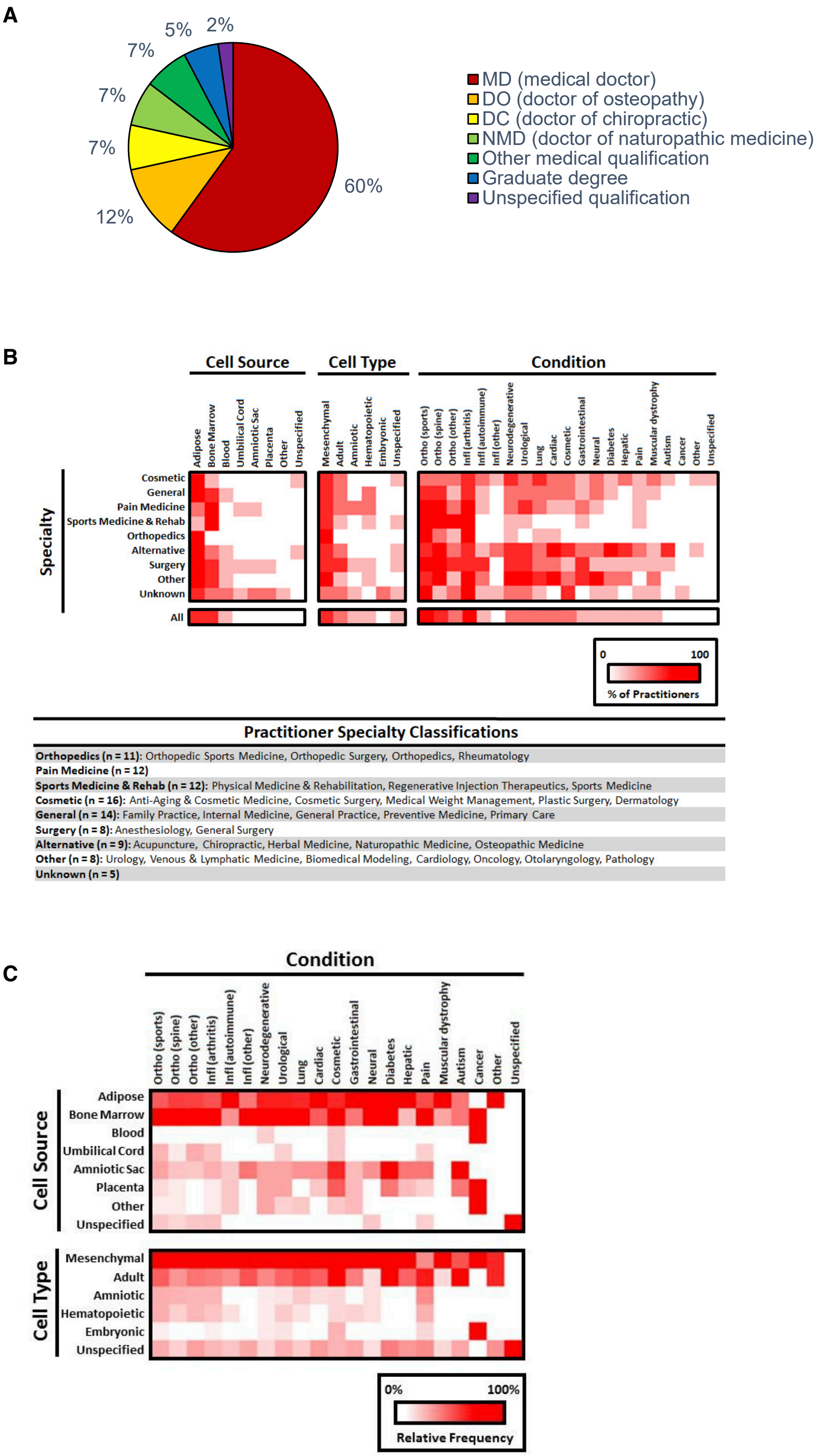

(legend on next page)

May 2019), and the number of stem cell clinics operating in the United States was estimated at 716 in May 2017 (Turner, 2018) and is likely to be even higher now. That 70%–80% of stem cell businesses are operating outside of franchises may limit the effectiveness of pursuing regulatory action by targeting franchises. This said, the FDA is currently seeking a permanent injunction against the Cell Surgical Network for numerous violations of good manufacturing and tissue practice, and for marketing products without FDA approval (United States of America v. California Stem Cell Treatment Center Inc, 2018), and previously won a court case against Regenerative Sciences for violating manufacturing and labeling requirements (United States of America v. Regenerative Sciences, LLC, 2014).

Intriguingly, approximately one-third (51/169) of stem cell businesses share a physical address with another medical or cosmetic clinic. These co-located businesses typically maintain different websites, phone numbers, and contact emails, but at least 40% of them share care providers (for the other 60% of clinics, the information supplied on the websites did not make it possible to identify whether there was overlap in care providers). The rationale for a care provider to maintain a stem cell business distinct from a medical practice is a matter of speculation, but this division may financially and legally insulate the two practices.

During the data collection process, we observed that businesses differ in the degree to which stem cell treatments are presented as central to their practice. Based on this, we subjectively divided businesses into one of three categories based on their degree of focus on stem cell treatments: stem cells as one treatment offered among many (37%), stem cells as a main treatment offered (38%), and stem cells as the sole focus of treatment (25%) (Figure 2B). The predominant model for stem cell treatments is thus not in the form of bespoke clinics, but rather as one type of treatment offered by businesses that may specialize in particular medical conditions (e.g., orthopedic conditions) or types of intervention (e.g., cosmetic surgery).

Sole-focus businesses do show some different patterns from businesses offering stem cell interventions as one of several types of treatment. For example, while 20% of stem cell businesses are part of franchises, this number reaches 38% for sole-focus businesses. Furthermore, 45% of sole-focus businesses are co-located with another medical practice.

Across the Southwest, two-thirds of clinics offer to treat more than one category of medical condition with stem cells. We identify a majority (71%) of clinics treating 1–3 categories of medical conditions, and only 1% that treat conditions from all of the 11 condition categories we identified (Figure 2C). Businesses focused solely on stem cells treat medical conditions across the full range of medical-condition categories. Of the 44 stem cell businesses in the Southwest that market treatments for four or more categories of medical conditions, those focused solely on stem cells are overrepresented (21/44, Figure 2C). Tracking the number of conditions treated by a given business can assist with identifying violations of FDA guidance on homologous use of human cells, tissues, and cellular and tissue-based products, which identifies the use of a single type of stem cell treatment for multiple medical conditions as an indication that the treatment might not adhere to the homologous use criterion (Food and Drug Administration, 2017).

Sole-Focus Businesses: Medical Expertise and Treatment Types

For clinics offering stem cells as one of many possible treatment options, it is typically impossible to determine from their websites which of the care providers might administer stem cell treatments. To examine the relationship between medical expertise and stem cell treatments, we narrowed our subsequent analysis to those businesses focused exclusively on stem cells. Of the 130 employees listed on the websites of sole-focus clinics in the Southwest, 60% have MD qualifications (Figure 3A). Other medical qualifications include Doctor of Osteopathy (12%), Doctor of Chiropractic (7%), and Doctor of Naturopathic Medicine (NMD, 7%).

Figure 3. Medical Expertise and Stem Cell Treatments

Data reflect sole-focus businesses only.

(A) Breakdown of employees by qualification (n = 130). "Other medical qualification" category includes Doctor of Podiatric Medicine (n = 1), physical therapists (n = 1), physician assistants (n = 5), and nurse practitioners (n = 2). Seven individuals listed non-medical graduate degrees, and a further three did not specify their qualifications. Two of the sole-focus businesses did not provide any information regarding practice employees or care providers.

(B) Relationship between practitioner specialty and stem cell source, stem cell type, and medical conditions treated. Data are shown as the percentage of practitioners with an indicated specialty who use a specific stem cell source or stem cell type as well as treat a specific medical condition.

(C) Relationship between stem cell source/type and medical condition treated. Data are shown as the column-normalized, relative frequency with which a specific condition is treated with a particular stem cell source/type. Analysis is based on information stated on stem cell business websites. A single business may use more than one cell type and cell source, and offer to treat more than one condition. Multiple care providers may also practice within a given business.

Interestingly, eight of the nine practicing NMDs in the Southwest are based in Arizona, and each received their NMD degree from the same naturopathic institution.

In addition to capturing the types of degrees held by care providers working in stem cell businesses, we worked to identify their medical specialties by systematically gathering their self-reported information regarding any board certifications and professional memberships. We observed that care providers of all specialties market the treatment of arthritis, spine, and sports injuries (Figure 3B). Specialists in orthopedics and sports medicine and rehabilitation were more likely to restrict stem cell treatments to those conditions related to their medical specialties (orthopedic conditions and arthritis). Providers listing specialties in cosmetic or alternative medicine were more likely to treat medical conditions across the full range of categories identified (with the exception of cancer).

As a whole, sole-focus businesses did not show different trends from the full set of businesses in the overall frequency of cell source or cell type used, or conditions treated (compare the "All" row in Figure 3B with Figures 1A–1C). Looking at the relationship between cell source and conditions treated, we identify that adipose tissue and bone marrow are used to treat the widest range of conditions (Figure 3C, upper panel). Across sole-focus businesses, any given condition appears to be treated using cells from multiple sources, and any given cell type is used to treat multiple conditions. Cosmetic, orthopedic, and inflammatory conditions are treated using cells from the widest range of sources, while treatments for muscular dystrophy are restricted to adipose and bone marrow sources. Similar patterns emerge when considering cell type rather than cell source (Figure 3C, lower panel), with mesenchymal and adult stem cells being used to treat every condition category. These heatmaps highlight the still highly experimental and diffuse landscape for stem cell treatments, with little convergence across clinics regarding which cell preparations might be best suited to treating different medical conditions.

DISCUSSION: POLICY IMPLICATIONS

Recent years have seen repeated calls for the FDA to take action against clinics marketing direct-to-consumer stem cell interventions (e.g., Turner and Knoepfler, 2016). Our analysis of stem cell businesses in the Southwest United States identifies several specific factors that could assist the FDA, state medical licensing boards, and prospective patients in prioritizing among businesses and care providers that warrant closer scrutiny.

First, we suggest that the 25% of stem cell businesses focusing exclusively on stem cell treatments be prioritized for closer attention. For clinics that offer stem cells as one of many treatment options, it can be difficult to identify which portion of their business is stem cell related.

Second, clinics purporting to use adipose tissue as a source of stem cells are also potential targets for scrutiny, as these procedures are likely to be out of compliance with the final FDA guidance adopted in 2017. With adipose tissue now classified as a structural tissue, clinics may be required to pursue FDA approval for treatments making use of this cell source. It remains to be seen whether stem cell clinics begin to move away from adipose tissue, and toward bone marrow, amniotic, or other stem cell sources, in light of this classification.

Third, we advocate greater scrutiny of those businesses offering to treat multiple condition types with stem cells. Approximately 30% of the clinics in the Southwest offer to treat four or more types of medical conditions, which may indicate a greater likelihood of violating FDA guidance on homologous use.

Fourth, the patterns we identify between medical expertise and conditions treated with stem cells can be leveraged to identify clinics for further scrutiny. Specialists in orthopedics and sports medicine and rehabilitation were more likely to restrict stem cell treatments to conditions falling within their specialty area. We identify specialists in cosmetic and anti-aging medicine as treating the widest variety of medical conditions, and suggest that practitioners with these specialties be prioritized for review by both FDA and state medical licensing boards. We also recommend that the helpful guide for patients published by the International Society for Stem Cell Research (2015) consider including a question about whether a practitioner's stated medical credentials are well suited to the medical condition for which a patient is seeking treatment.

In summary, our detailed characterization of stem cell businesses in the Southwest United States offers new insights into the practices of direct-to-consumer stem cell clinics, and identifies a concrete set of variables that may be of assistance to regulatory bodies and patients trying to make sense of this rapidly growing market in the United States.

EXPERIMENTAL PROCEDURES

We gathered publicly available, online material relating to stem cell businesses and their practitioners, including information relating to the types and sources of stem cells used, the conditions treated, the franchise status of the business, and the self-reported qualifications of their medical providers. We began by characterizing clinics already identified by Turner and Knoepfler (2016), who listed 128 businesses operating across 207 locations in the six Southwest states. By expanding our internet search terms to include US State names and large cities in the Southwest, we

identified an additional 41 businesses and 33 clinic locations in these six states, for a total of 169 businesses across 238 clinic locations (Table S2). We do not claim to have identified a comprehensive list of clinics operating in the Southwest, but our findings suggest that the estimate by Turner and Knoepfler (2016) was conservative, and/or that the number of clinics increased significantly between the end of their data collection (February 2016) and ours (August 2017). Indeed, a revised estimate from Turner (2018) suggests 716 clinics operating across the United States as of May 2017, compared with 570 in February 2016.

There is great heterogeneity in the terminology and type of information presented on the websites of stem cell businesses. To this end, we developed a uniform classification system and categorization of stem cell types and sources, medical conditions, and practitioner specialties presented across the websites. Table S1 groups all medical conditions treated by stem cell businesses into a set of broader categories. An overview of all the data collected is presented for each business, in de-identified form, as Table S3.

SUPPLEMENTAL INFORMATION

Supplemental Information can be found online at https://doi.org/10.1016/j.stemcr.2019.07.001.

AUTHOR CONTRIBUTIONS

Project Conceptualization and Supervision, E.K.F. and D.A.B.; Data Collection, A.M., E.K.F., L.K., P.W., B.B., J.P.N., and A.P.; Data Curation and Analysis, A.M. and L.K.; Writing – Original Draft, A.M. and E.K.F.; Writing – Review & Editing, D.A.B. and E.K.F.; Figure Preparation, A.M., D.A.B., and E.K.F.

ACKNOWLEDGMENTS

E.K.F. acknowledges funding from the Lincoln Center for Applied Ethics and the Institute for Social Science Research at Arizona State University.

Received: March 15, 2019
Revised: June 28, 2019
Accepted: July 1, 2019
Published: August 1, 2019

REFERENCES

Daley, G.Q., Hyun, I., Apperley, J.F., Barker, R.A., Benvenisty, N., Bredenoord, A.L., Breuer, C.K., Caulfield, T., Cedars, M.I., Frey-Vasconcells, J., et al. (2016). Setting global standards for stem cell research and clinical translation: the 2016 ISSCR Guidelines. Stem Cell Reports *6*, 787–797.

Food and Drug Administration. (2017). Regulatory considerations for human cells, tissues, and cellular and tissue-based products: minimal manipulation and homologous use; guidance for industry and Food and Drug Administration staff, Federal Register. Retrieved from. https://www.federalregister.gov/d/2017-24838.

International Society for Stem Cell Research. (2015). Patient Handbook on Stem Cell Therapies (International Society for Stem Cell Research), Retrieved from. http://www.closerlookatstemcells.org/docs/default-source/patient-resources/isscr-patient-handbook-english_ltr_17nov2016_web-only.pdf?sfvrsn=2.

Knoepfler, P.S. (2017). The stem cell hard sell: report from a clinic's patient recruitment seminar. Stem Cells Transl. Med. *6*, 14–16.

Knoepfler, P.S., and Turner, L.G. (2018). The FDA and the US direct-to-consumer marketplace for stem cell interventions: a temporal analysis. Regen. Med. *13*, 19–27.

Lau, D., Ogbogu, U., Taylor, B., Stafinski, T., Menon, D., and Caulfield, T. (2008). Stem cell clinics online: the direct-to-consumer portrayal of stem cell medicine. Cell Stem Cell *3*, 591–594.

Rachul, C., and Caulfield, T. (2015). Gordie Howe's stem cell "miracle": a qualitative analysis of news coverage and readers' comments in newspapers and sports websites. Stem Cell Rev. *11*, 667–675.

Sipp, D., Caulfield, T., Kaye, J., Barfoot, J., Blackburn, C., Chan, S., De Luca, M., Kent, A., McCabe, C., Munsie, M., et al. (2017). Marketing of unproven stem cell-based interventions: a call to action. Sci. Transl. Med. *9*. https://doi.org/10.1126/scitranslmed.aag0426.

Srivastava, A., Mason, C., Wagena, E., Cuende, N., Weiss, D.J., Horwitz, E.M., and Dominic, M. (2016). Part 1: defining unproven cellular therapies. Cytotherapy *18*, 117–119.

Turner, L., and Knoepfler, P. (2016). Selling stem cells in the USA: assessing the direct-to-consumer industry. Cell Stem Cell *19*, 154–157.

Turner, L. (2018). The US direct-to-consumer marketplace for autologous stem cell interventions. Perspect. Biol. Med. *61*, 7–24.

United States of America v. California Stem Cell Treatment Center Inc. (2018). United States District Court for the Central District of California Eastern division, 5:18-CV-1005.

United States of America v. Regenerative Sciences, LLC (2014). United States Court of Appeals for the district of Columbia circuit, 12-5254.

Stem Cell Reports, Volume *13*

Supplemental Information

Characterizing Direct-to-Consumer Stem Cell Businesses in the South-west United States

Emma K. Frow, David A. Brafman, Anna Muldoon, Logan Krum, Paige Williams, Bryson Becker, John P. Nelson, and Ashley Pritchett

Supplemental Information

Table S1. List of medical conditions treated with stem cells across the businesses characterized (related to Fig.1C).

**Condition Category**	**Conditions included in the category**
Autism	
Cancer	Breast cancer
	Prostate cancer
Cardiac	Cardiovascular problems
	Chronic heart failure
	Ischemic cardiomyopathy
	Ischemic stroke
	Myocardial infarction
	peripheral artery disease
	refractory angina
Cosmetic	anti-aging
	breast augmentation
	breast reconstruction
	buttock augmentation
	cutaneous photo-aging
	Facelift
	facial rejuvenation
	hair rejuvenation
	Skin
Diabetes	
Gastrointestinal (GI)	Crohn's disease
	ulcerative colitis
Hepatic	fatty liver / liver insufficiency
	Hepatitis
	auto-immune hepatitis
Inflammatory	SI joint inflammation
	Facet joint syndrome
(sub-category) Arthritis	basal joint arthritis
	carpometacarpal arthritis
	degenerative osteoarthritis
	Osteoarthritis
	rheumatoid arthritis
	spinal arthritis
	sub-talar arthritis
(sub-category) Autoimmune	Alopecia

	Lupus
	relapsing polychondritis
Lung	Asthma
	COPD
Muscular dystrophy	
Neural	cognitive impairment
	CIDP
	diabetic neuropathy
	Dysautonomia
	myasthemia gravis
	occipital neuralgia
	optic neuritis
	peripheral neuropathy
	Radiculopathy
	Sciatica
	Stroke
	traumatic brain injury
Neurodegenerative conditions	ALS
	Alzheimer's
	cerebral palsy
	Huntington's
	Multiple sclerosis
	Parkinsons
Orthopedic	AC joint separation
	avascular osteonecrosis
	Baker's cyst
	Bursitis
	Bunion
	carpal/tarsal tunnel syndrome
	Dupuytren's contracture
	Osteoporosis
	pinched nerve/radiating pain
	plantar fasciitis
	spinal muscular atrophy
	Spur
	thoracic outlet syndrome
	Tumor
(sub-category) Sports injuries	Achilles injuries

	ACL
	Fractures
	Golfer's elbow
	Instability
	Meniscus
	osteochondral lesions
	patellar, patellofemoral syndrome
	post-surgery
	recurrent dislocations
	rotator cuff tendonitis
	runner's knee
	Sclerodoma
	Sprains
	tendonitis, tendonopathy
	tennis elbow
	TFCC tear
	trigger finger
(sub-category) Spine	annular tear
	Deformity
	degenerative disk disease
	failed back surgery
	herniated disk
	Scoliosis
	spondylosis; spondylolisthesis
	spinal stenosis
	Stenosis
Pain	General
	headaches/migraines
	post-surgery
	Whiplash
Urological	erectile dysfunction
	interstitial cystitis
	peyronies disease
	bladder conditions
	kidney conditions

Table S2. Comparison of number of stem cell businesses & clinics identified by Turner and Knoepfler (2016) and those identified in the current study (related to Experimental Procedures).

	**Current study**		**Turner & Knoepfler (2016)**	
**State**	**Businesses**	**Clinics**	**Businesses**	**Clinics**
**Arizona**	34	47	27	36
**California**	79	105	71	113
**Colorado**	28	41	19	37
**New Mexico**	6	8	1	2
**Nevada**	11	15	5	7
**Utah**	11	22	5	10
**TOTAL**	169	238	128	205

Table S3. Data summary for direct-to-consumer stem cell businesses in the Southwest US (related to Experimental Procedures). State locations are provided for each business, but specific business names are not identified.

See Excel file for Table S3.

Supplemental Experimental Procedures

Online data collection

Stem cell businesses were identified through state-by-state internet searches between June 2016 and August 2017. The list of businesses provided by Turner and Knoepfler (2016) was used to create an initial list, and was then expanded through independent searches for businesses in specific states and regions in the Southwest US. Specific search terms used are listed at the end of this document.

Information was collected from the public sections of stem cell business websites and inputted into an Excel spreadsheet. For each characterized stem cell business, screenshots were taken of each webpage that mentioned stem cells (using Snagit software), to construct an archive for future reference. After the initial data collection, a randomized 25% of the clinics characterized by each researcher was cross-checked by a different member of the research team, to ensure consistency in data collection practices across researchers. Any discrepancies in data collection were flagged and discussed collectively, and a consensus data entry agreed upon.

The data collected reflects the public presentation of a stem cell business; it is assumed to be accurate, but is not guaranteed to represent the actual practices of a given business. For example, a given clinic might not list all the conditions they treat with stem cells, or might not in practice treat all the conditions that they list online.

Condition Categories

A complete list of medical conditions treated with stem cells was compiled all from stem cell business websites characterized, and the research team worked collectively to collapse this list into the 11 broad categories listed in Table S1.

Medical Specialties

The care providers listed for each stem cell business were copied into the data collection spreadsheets, together with any specialty and/or board certification information listed on the business website. For any providers who did not list information regarding their professional qualifications, Google searches were undertaken to identify any specialties, board certifications, or professional association memberships. If a given practitioner made information available on a university or other clinic website, this was used. For those who did not, searches were conducted to identify available biographies from conferences, fellowships, or professional societies. If no listing for a care provider could be found or no names were provided on a clinic's website, the provider was listed as of "unknown" expertise.

Once identified, medical specialties were grouped into related categories for analysis (see Fig. 3B). Some specialties were combined for brevity. For example, "anti-aging," "anti-aging and cosmetic medicine," and "cosmetic medicine" were combined into "anti-aging and cosmetic medicine". "General practice" and "general medicine" were combined into "general practice."

Stem Cell Business Search Terms

Stem cell clinics Arizona
Stem cell treatment clinic Southern California
Northern California stem cell treatment center
Norcal regenerative cell treatment
Bay Area stem cell treatment center
Colorado stem cell clinics
Stem cell clinics AND New Mexico
Stem cell clinics and NM
Stem cell clinics in New Mexico
Adipose tissue AND New Mexico
Stem cell clinics Nevada
Stem cell clinics of Nevada
Small stem cell clinics in Nevada
Stem cell clinics Utah
Health AND stem cells
